# Multicenter Study Evaluating Impact of Patient and Sonographer Demographics on Quality of Focused Cardiac Ultrasounds

**DOI:** 10.5811/westjem.38462

**Published:** 2025-10-03

**Authors:** Barret Zimmerman, Tracy E. Madsen, Giorgina Giampaolo, Jennifer Rogers, Hilary Davenport Stroud, Creagh Turner Boulger, Michael I. Prats, Alice Wu, Megan Leo, Joseph R. Pare, Meera Muruganandan, Josh Kaine, Daniel S. Brenner, Pam Cruz Soriano, Nadia Aracelliz Villarroel, Michele L. Schroeder, Natalie Strokes, Anna Tyson, Timothy Gleeson, Michael Hill, Janette Baird, Alyson J. McGregor, Kristin H. Dwyer

**Affiliations:** * Harvard Medical School/Mass General Brigham, Department of Emergency Medicine, Boston, Massachusetts; † Alpert Medical School of Brown University, Department of Emergency Medicine, Providence, Rhode Island; ‡ The Ohio State University, Department of Emergency Medicine, Columbus, Ohio; § Boston University Chobanian & Avedisian School of Medicine, Department of Emergency Medicine, Boston, Massachusetts; ¶ Baystate Medical Center, Department of Emergency Medicine, Springfield, Massachusetts; || Indiana University School of Medicine, Department of Emergency Medicine, Indianapolis, Indiana; ** UMass Chan Medical School, Department of Emergency Medicine, Worcester, Massachusetts; †† University of South Carolina School of Medicine/Prisma, Department of Emergency Medicine, Greenville, South Carolina

## Abstract

**Introduction:**

Demographic inequities in cardiovascular care have been well established, with evidence of effects from sex, age, and body mass index (BMI). For instance, women are less likely to receive guideline-based care for acute myocardial Infarction, bystander cardiopulmonary resuscitation, or recognition of cardiac arrest. We investigated the impact of patient sex, along with other patient demographics such as age and BMI, on the quality of focused cardiac ultrasounds (FOCUS). We hypothesized that females would have lower overall FOCUS quality and more frequently omitted apical four-chamber (A4C) views due to breast tissue. Secondary objectives included evaluating differences in image quality and omission rates by BMI, and by age and sonographer sex and training level.

**Methods:**

In this multicenter, retrospective study we investigated 1,200 total adult patients (100 females and 100 males per site) at six participating sites. The FOCUS quality was determined by two blinded experts per site using a 1–5 ordinal scale per view (parasternal long, parasternal short, A4C, and subxiphoid). The primary outcome, overall quality, was the summed score of the four views, with a maximum score of 20. This scale was then collapsed into three categories for the individual FOCUS views: images inadequate to support diagnosis; images meeting the minimum to support diagnosis; and images supporting the diagnosis well. Secondary outcomes were A4C quality and omission rate. We evaluated associations between sex and FOCUS overall quality using unadjusted mixed-effects models followed by multivariable mixed-effects models adjusted for patient age, BMI, operator sex, and operator experience level.

**Results:**

The A4C images of female patients were of significantly lower quality (P < .001) and had been omitted more frequently (P < .001); male patients had > 60% higher odds of a diagnostic A4C view (95% CI 1.3 – 2.0). Overall FOCUS quality decreased as BMI deviated from normal and as age increased. There was no significant difference in overall FOCUS quality between female and male patients.

**Conclusion:**

We did not find sex-based differences in overall FOCUS quality; however, we did find that females received lower quality apical four-chamber views and had this view omitted more frequently. Additionally, overall quality declined as BMI deviated from normal, and as age advanced. Future research should elucidate the clinical implications of these differences in quality and the explanation behind not obtaining high-quality views in older patients, in individuals whose BMI deviated from normal toward either underweight or overweight, or in female patients.

## INTRODUCTION

Heart disease is the leading cause of death in the United States and worldwide, and cardiopulmonary complaints are among the most frequent presentations to the emergency department (ED).^1^ Demographic discrepancies in cardiovascular care have been established, and patient sex has emerged as a significant factor. For example, women suffer greater mortality from myocardial infarction but are less likely to receive guideline-based pharmacological therapies or revascularization for acute myocardial infarction.^2^ Other sex disparities are evident along the trajectory of care in the ED from diagnosis to management, and women may experience higher rates of short-term mortality and adverse events.^3^ It has also been shown that women are less likely to receive bystander cardiopulmonary resuscitation and automated external defibrillator (AED) placement for witnessed out-of-hospital cardiac arrest, which is believed to be due to lack of recognition of this emergency in women as well as hesitancy in exposing a woman’s chest.^4^,^5^

Besides sex, other demographic factors have also been shown to significantly impact care. For instance, patient age seems to have profound impacts on care, and older adults have been understudied as a group.^6^ The importance of studying the relationship of patient demographic factors to cardiovascular care is underscored when considering patient weight, where a U-shaped relationship has been discovered. While underweight patients have worse outcomes and the most obese patients also do poorly, overweight or obese patients have exhibited better outcomes (the so-called “obesity paradox”).^7^

To our knowledge, no study has evaluated whether these disparities extend to focused cardiac ultrasound (FOCUS) in the ED. Point-of-care ultrasound is a fundamental aspect of modern emergency care^8^ and may have a particularly powerful role in changing management and outcomes in cardiopulmonary complaints by reducing morbidity, mortality and ED length of stay.^9^,^10^ One study evaluating sex-based differences among comprehensive echocardiograms actually found that men received more non-diagnostic studies.^11^ However, comprehensive echocardiograms are performed in a vastly more controlled setting, whereas ED FOCUS are often performed on patients wearing street clothing who are unstable with limited mobility and sometimes in very public spaces such as the hallway. We hypothesized that sex trends for FOCUS would be more aligned with the acute myocardial infarction and resuscitation literature, ie, more non-diagnostic FOCUS for female ED patients. The impact on FOCUS quality of other patient characteristics, such as body mass index (BMI) or age, is similarly unknown. Body mass index seems to affect a clinician’s approach to and interpretation of other forms of cardiopulmonary imaging, such as chest radiography,^12^ but to our knowledge the effect of BMI on FOCUS quality has not been investigated.

**Figure 1:**
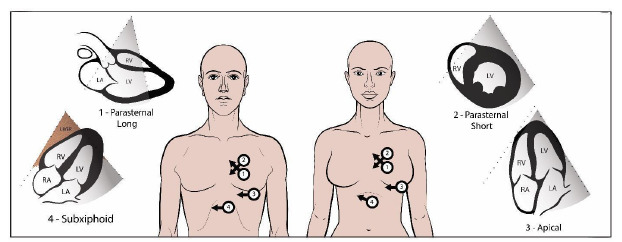
Probe positioning to obtain the four focused cardiac ultrasound views on a male and a female patient.

Demographic factors such as patient sex, BMI, and age affect the composition and arrangement of body tissues, which may affect ultrasound image quality. The FOCUS consists of four primary views of the heart, each view contributing different clinical information, and no single view provides complete diagnostic information^13^ (Figure 1). The apical four-chamber (A4C) view is obtained just under the pectoralis muscle in the fourth or fifth intercostal space at the apical impulse and provides vital diagnostic information regarding ejection fraction, valvular disease, right heart strain, and diastology. In women, this is often within the breast crease. The positioning required for this view could be particularly susceptible to anatomic and social influences on image acquisition.

**Population Health Research Capsule**

What do we already know about this issue?

*Demographic factors (sex, weight, etc) affect cardiac care, including imaging, but little is known about their impact on cardiac ultrasound, especially in the ER.*

What was the research question?

*Do demographic factors affect overall cardiac ultrasound imaging quality and omission of views in the ED?*

What was the major finding of the study?

*Females had lower Apical 4-Chamber (A4C) quality and more A4C omissions (p<0.001); overall image quality was the same.*

How does this improve population health?

*Demographic factors are associated with important and clinically significant differences; future education, design, and research should focus on these discrepancies.*

We postulated that clinician or patient discomfort with breast exposure and image-acquisition challenges unique to women may correlate with differences in clinician behavior and image quality. Establishing this relationship could help build a framework for quality improvement in FOCUS, which could support more accurate diagnosis and management of highly morbid conditions. Because this is a relatively unstudied topic, a large, multicenter study, evaluating existing patterns would help elucidate the status quo and prime what will hopefully be a fruitful area of research and intervention. Elucidating other areas where inequity occurs could inspire new educational interventions, modified techniques, or equipment innovations.

## METHODS

### Study Design and Setting

This study was a multicenter, retrospective chart review^14^ of ED patients who received a FOCUS between January 2018–January 2021 at six academic Level 1 trauma center health systems in the United States. Annual ED volumes at the primary sites ranged from 95,000–130,000. Each site has a residency training program and an active emergency ultrasound education program for residents and fellows. The institutional review boards at each center approved this study. As per chart review best practices^14^ and to reduce bias, the blinded and trained abstractors were without bias and performed a systematic chart abstraction. The data abstracted was limited to basic demographics and was available in the chart. Missing data was not a significant issue.

### Selection of Participants

Eligible for inclusion were all adult patients (≥ 18 years of age) with a FOCUS saved in each study site’s image storage solution (Qpath-e, Maple Ridge, BC, Canada; Ultralinq, Athlone, Ireland; or Synchronicity, Bothell, WA ) during the study period and submitted for quality assurance. The FOCUS studies must have been performed by a physician operator (eg, resident, fellow, or attending), and operator experience level was determined by the highest level of training obtained by the physician listed as the operator in the storage solution software. (Often, more than one operator is listed as performing the study.) Each of the six sites identified 100 female and 100 male consecutive patients (1,200 total) who underwent FOCUS (Table 1a). Based on our primary outcome of overall FOCUS quality, our sample size of 200 per site was based on an a priori power calculation so that each site would be able to detect a two-point sex difference in the 20-point scale (β = 0.80, two-tailed ɑ = 0.05). Each site determined an enrollment start date within the data collection period, (or two start dates if dividing winter/summer, which sites were allowed to do) and identified consecutive patients in the image storage system who received a FOCUS until they had enrolled 100 female and 100 male patients. The data collection period spanned three years across all sites combined; however, for each individual site, the data collection period was a small fraction of that time. One trained individual at each site performed chart abstraction of deidentified patient demographic data such as age, BMI, and sex using a standardized data collection form. (Appendix 1) This individual was not one of the physicians scoring the FOCUS image quality.

### Measurements

At each site, the two faculty members (ultrasound fellowship trained) performing quality review were blinded to the patient’s health record and to the other reviewer’s scoring. Reviewers scored each of the available four views (Figure 1): parasternal long axis; parasternal short axis; A4C; and subxiphoid. Scoring criteria ranging from 1–5 per view on an ordinal scale with 5 being highest (Table 2) Omitted FOCUS views were noted on the spreadsheet. The two expert reviewers at each site scored the FOCUS exams independently using the same scoring rubric, and their scores were subsequently averaged. This rubric is suggested for use by an American College of Emergency Physicians clinical guideline.^15^

### Statistical Analysis

Data collected from the six study sites were imported into SAS v9.4 (SAS Institute Inc, Carey, NC) for analysis. We investigated three outcome variables through separate mixed-effects regression models: overall FOCUS quality; A4C quality; and A4C omission. Our primary outcome was overall FOCUS quality, ie, the summed score of the four FOCUS views. Our secondary outcome measures were the individual FOCUS scores and omission rates. We aimed to evaluate the impact of patient and sonographer demographics on the FOCUS scores and omission rates. Other than patient sex, the demographics of interest were BMI (normal = 18.5–24.9 kg/m2, overweight = 25–29.9 kg/m2, and obese = 30.0+kg/m2), age, sonographer sex, and sonographer training level.

#### Overall Quality of Focused Cardiac Ultrasound

The overall FOCUS quality was treated as a continuous outcome variable from 1–20 summed across the four views. The scores of the two raters per site were averaged to create one patient score for the site. First, we performed an unadjusted, mixed-effects linear regression model to determine the association between overall quality and patient sex, with study sites considered as random effects. The models were then adjusted for patient age, BMI, operator sex, and operator experience level. We tested interaction terms between patient sex and operator sex. Mean differences, 95% confidence intervals (CIs), and *P*-values were reported. *P* < .05 was considered to be statistically significant.

#### Quality of Apical Four-Chamber Views

For A4C quality, there were a limited number of FOCUS views with scores of 5. To address statistical modeling issues due to small cell sizes for scores of 5, we collapsed the 1–5 scores into three clinically relevant categories: category 1 comprised scores of 1–2 (poor image quality insufficient for diagnosis); category 2 comprised scores of 3 (adequate but with flaws that could affect interpretation); and category 3 was comprised of scores of 4–5 (high quality images that allowed for a confident answer to the clinical question being asked of that view). We ran unadjusted, mixed-effects logistic regression models to evaluate the association between A4C quality and patient sex (A4C quality of 2 vs 1 and A4C quality of 3 vs 1 as the outcomes); study sites were included as random effects terms. We then adjusted models for patient age, BMI, operator sex, and operator experience level. As above, interaction terms between patient sex and operator sex were tested. We reported odds ratios with 95% CI.

#### Interrater Agreement Assessment

We calculated weighted kappas to evaluate the rating agreement between the two reviewers for each of the four views. Additionally, we used Kendall tau coefficients to measure the ordinal association between pairs of ratings.

## RESULTS

### Characteristics of Study Subjects

A total of 1,200 patients (50% female, 50% male) were included across six sites during the study period (January 2018–January 2021). Table 1a shows the demographic characteristics of the study sample. A significant portion of study participants were > 65 years of age (Table 1). The largest proportion of participants had a calculated BMI within normal range, followed by overweight (Table 1). Most sonographers were resident physicians, and a slight majority identified as male (Table 1b).

### Image Quality and Omission Rates

Female patients had lower average A4C quality scores than male patients and were more likely to have the A4C view omitted, *P* < .001. However, male and female patients had similar overall FOCUS quality (Table 3b). Both the unadjusted and adjusted mixed-effects linear regression models showed no significant difference in total FOCUS score for female vs male patients (Appendix 3). Females had slightly higher PSS scores, and lower omission rates (Table 3a/b). The patient sex by sonographer sex interaction term was not significant.

Patient sex was statistically significant in both the unadjusted and adjusted mixed-effects logistic regression models for the A4C quality outcome (Appendix 3). Unadjusted, male patients had higher odds of receiving a diagnostic A4C view compared to female patients (Appendix 3).

### Sonographer Demographics

Scans performed by male sonographers, on average, scored lower than those performed by female sonographers (Appendix 3). Scans with attending sonographers had lower overall FOCUS quality scores than scans with resident sonographers (Appendix 3). Scans with male sonographers were less likely than scans with female sonographers to achieve an adequate A4C score (Appendix 3).

### Body Mass Index and Quality of Focused Cardiac Ultrasound

**Figure 2:**
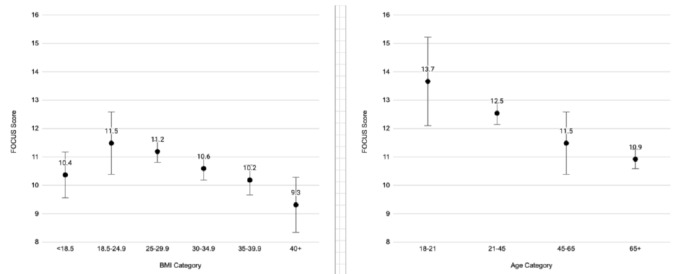
Total focused cardiac ultrasound score by (a) body mass index category (ref 18.5–24.9) and (b) age category (45–65). Bars indicate 95% CI.

Underweight and obese patients had lower total FOCUS quality scores compared to patients with normal BMI (Figure 2a). Total FOCUS quality scores decreased with deviation from normal BMI. Both obesity and being underweight were negatively associated with A4C score. Patients with an obese BMI had 62% lower odds of receiving a high-quality A4C compared to patients with a normal BMI, and this difference became more pronounced as weight increased to the highest weight category (BMI 40+) (Appendix 3).

### Age and Quality of Focused Cardiac Ultrasound

On average, our youngest age category had higher total FOCUS scores compared to middle-aged patients (Figure 2b). Quality of FOCUS decreased with advancing age (Figure 2b).

### Interrater Agreement

On average, there was 30%–60% agreement on ultrasound scores between the two reviewers on the five-point scale ratings. Furthermore, the ratings between the two reviewers had moderate (kappa minimum = 0.49) to strong (kappa maximum = 0.70) positive agreement, and the Kendall tau also showed the range of rater concordance to be moderate (0.34–0.68).^16^

## DISCUSSION

We investigated whether there were discrepancies in FOCUS quality correlating with patient and sonographer demographics. In this large, retrospective, multisite study, A4C views on female patients were of lower quality and more often omitted, and male sonographers were more likely to achieve non-diagnostic A4C images; however, we found no significant difference in overall FOCUS quality for male compared to female patients. Although the absolute effect size of some of these differences was small (eg, 2.3 vs 2.6 score for A4C view on five-point scale for females vs males), that females were 30% less likely to receive a diagnostic A4C view that could have been clinically significant. The fact that we found differences in the A4C view but not in overall quality could signal that clinicians may be attempting to use alternative views to compensate for and obtain similar information among the sexes. Supporting this notion is that parasternal short axis scores for females were of slightly higher quality and less frequently omitted. Our trial was not designed to discern compensatory behavior.

The A4C view provides unique information, and we believe its omission has important clinical implications. If avoiding the A4C view in females is related to exposure of the chest, this would be in line with research on cardiopulmonary resuscitation and AED placement establishing that female chest exposure has been an impediment to ideal care, especially in public settings.^5^ This has further implications as “hallway medicine” becomes more commonplace in EDs. Given the potentially sensitive position in which the operator’s hand and probe must be placed to obtain the view—exposing the chest—and that breast tissue could obfuscate this view, it is not surprising that female patients had fewer diagnostic A4C views, and that male sonographers in particular are 30% more likely to obtain a non-diagnostic view.

Part of this discrepancy may also relate to biases inherent in training materials. For example, live ultrasound models and ultrasound simulation diagrams or manikins traditionally feature prototypical male anatomy. We should work to incorporate women into our ultrasound education and normalize examination of female anatomy in a patient-centered way.

Future research could elucidate the etiologies of these discrepancies. Whether they are social, anatomical, or environmental has implications for addressing them: if differences are predominantly due to social factors, clinician educational interventions could be instituted and paired with a dialogue script to facilitate communication and ease of the exam, similar to the way in which other sensitive examinations (eg, pelvic) are taught. If anatomy is found to be the larger factor, innovations that alter the chain between the patient’s body position, probe, hand, and operator could be engineered to address this. Finally, environmental effects, although sometimes beyond the control of individual clinicians, could reinforce the value of private rooms, privacy screens, or gowned patients.

As a patient’s BMI deviated from normal toward either underweight or overweight their FOCUS quality scores decreased (Figure 2a). Patients with a normal BMI had the highest overall quality FOCUS scores, suggesting that either sparse or abundant subcutaneous tissue presents challenges to obtaining high-quality views. Underweight individuals may be difficult to scan due to a lack of tissue to seat the probe between the ribs. This discrepancy is reminiscent of the “U-shaped” relationship that has been demonstrated regarding BMI and overarching cardiovascular clinical outcomes. In this case, patients with higher BMI may have additional tissue that obscures landmarks, creates a physical barrier to probe positioning, and introduces more tissue between the probe and the target organ. Higher BMI patients may also not be as physically able to cooperate with body positioning maneuvers to optimize the A4C view. Patient age also significantly impacted total FOCUS quality, with deterioration as age increased (Figure 2b). There are many possible reasons why younger patients might receive higher quality scans. These may include a greater ability to easily lie flat or on their left side, a lower acuity allowing for more time to perform the exam, a lower likelihood of receiving positive pressure ventilation, or a higher percentage of educational scans. Future work specifically targeting the underweight, overweight, and advanced-age populations should focus on optimizing technique and equipment (probes, sensors, software).

Clinician sex and training level are correlated with statistically significant changes in overall FOCUS quality. Although male physicians produced lower quality images, the difference was minor, and it is unclear what may have led to this outcome. Previous studies have found inequity in cardiovascular care between sexes that was modified by the sex concordance of the clinician and patient^17^; however, in our sample, clinician-patient sex interactions were not significant. Attending physicians, as compared to residents, produced lower quality images, which most likely reflects the positive influence of contemporary medical education on ultrasound skills. As with other procedures, skill attrition may occur over time. Further studies should explore this relationship to optimize continuing education for attendings.

## LIMITATIONS

This retrospective study could not account for the clinical scenario in which FOCUS was used. As a result, the information sought with FOCUS by the operator was uncertain; it is possible that the necessary clinical information in some studies was attainable without an A4C view and, therefore, it was deemed unnecessary by the clinician. It is also possible that some individual views or studies were never saved due to poor windows, and the retrospective design prevented us from knowing to what extent this happened in various groups. In our best attempt to account for this lack of context, the primary outcome of total FOCUS quality was a summed score reflecting the total amount of clinical information the operator obtained. It is also possible that some studies were never saved at all due to poor views, and the retrospective design prevented us from determining to what extent this happened in various groups. Additionally, we acknowledge that BMI reporting may not always have been accurate given that ED patients do not always have updated weights and heights measured in the ED due to the acuity of care and variability in availability of equipment.

A further limitation is that we included some educational scans in our study due to the heterogeneous nature of ultrasound logging culture at different institutions. In theory, educational scans may show biases toward young, healthy, compliant, normal BMI, and male patients, a population to which learners may be drawn to select for the perceived ease of obtaining images. To minimize this, we interviewed each site regarding their cultural practices and the expected composition of their scans and concluded that most “educational” scans were clinically indicated scans performed on patients with acute pathology. Therefore, we feel that the data presented is representative of clinical FOCUS use at academic institutions.

We controlled for operator experience level in our adjusted model, and by finding an impact of operator experience level it seems logical that within our experience-level groups there was further experienced-based heterogeneity. The granularity of those differences could not be captured by our data. For instance, a junior resident may not obtain as high-quality images as a senior resident, and a recently trained attending may outperform an attending who was trained decades ago without formal ultrasound training.

Kappas in our study were moderate. The scale we used is, as far as we know, the most ubiquitous FOCUS scale that has been suggested for use by an ACEP clinical guideline^15^ and has been the default scale for quality assessment software. Despite this, it is unstudied, and its reliability and validity still need to be determined. As far as we know, our study is the first to explore point-of-care ultrasound quality quantitatively as it relates to patient sex, clinician sex, and other demographic factors. Previous literature in cardiology discusses diagnostic or non-diagnostic scans but did not use a quantitative score. For all categorical ordinal data, we chose a priori reference groups, and for each demographic we used our best judgment in selecting the a priori reference group.

Due to small cell sizes for a score of five, we condensed the individual view scores down to three categories for this separate analysis. However, the total score of 20 remained (four views with a possible score up to five for each view). This could have impacted results by failing to capture differences among the extreme ends of the scale. To minimize this, we selected categories that were overall clinically very similar (For example, grouping “no recognizable structures” with “minimally recognizable structures - insufficient for diagnosis” created a category in which none of the images were good enough to draw a diagnostic conclusion.) Scores of 4 or 5 are also very similar, and both easily support a diagnosis.

Finally, the allowed data collection period spanned three years across all sites combined; however, the identified echocardiograms for inclusion at each individual site occurred within the period of a a few months. The volume of FOCUS at each site was high, and 200 consecutive FOCUS scans were easily identified in a fraction of the full study period. Each site began their data collection at various times based on IRB approval and changes in image storage solutions. We do not believe that the ultrasound technology advanced significantly during this time frame. Neither do we believe that images taken earlier in the study period were of inherently lower quality than those obtained later in the study period due to technological advancements.

## CONCLUSION

While we found that females received lower quality images and more frequently omitted apical four chamber views on their focused cardiac ultrasound, we did not find a difference in overall FOCUS quality by sex. The A4C view provides information about the right ventricle and valves that cannot be obtained from other views. Females were significantly less likely to receive a diagnostic A4C view, and we believe that even small differences in data acquisition from the A4C view could represent a clinically important finding. Another important and clinically significant finding was that underweight and overweight patients receive lower quality images than patients with a normal BMI. Perhaps for our underweight patients we can apply a gel pad to better optimize their images.

Older patients, for whom cardiovascular disease is more common, also received lower quality FOCUS scores. It is of interest that male sonographers and attending physician sonographers both achieves lower quality scores. While it is unclear why male sonographers’ image quality was worse, the difference was slight. However, for older attendings, perhaps we were witnessing skills attrition vs attendings who graduated before ultrasound was core to resident education. Skills attrition is not unique to ultrasound.

Our study, which investigated some of the most important demographic factors and how they may affect FOCUS image acquisition and quality, represents an essentially unstudied aspect of a core modality in our specialty. It is also, to our knowledge, the first study to report kappas on a widely used quality assessment metric—the five-point echo scale used by many institutions for internal quality review of FOCUS images. Further studies should attempt to validate this quality scale and prospectively examine the relationship between demographic factors and ultrasound image quality. Ultimately, we should seek to explore the implications and rationale behind the tendency to omit or be unable to obtain a high-quality view and the clinical rationale for performing or not performing views based on patient presentation.

## Supplementary Information







## Figures and Tables

**Table 1a:** Baseline Characteristics of Patients

	n	%
Sex		
Male	600	50
Female	600	50
Total	1200	
Gender		
Men	600	50
Women	600	50
Total	1200	
Age		
18–20	14	1.2%
21–44	264	22.1%
45–64	440	36.8%
65+	479	40.0%
Total	1197	
BMI		
<18.5	39	3.6%
18.5–24.9	316	29.2%
25–29.9	298	27.5%
30–34.9	226	20.9%
35–39.9	104	9.6%
40+	100	9.2%
Total	1083	

BMI, body mass index.

**Table 1b:** Baseline Characteristics of Sonographers

	n	%
Sex		
Men	652	54.6
Women	542	45.4
Total	1194	
Level of Training		
Attending	138	11.6
Fellow	57	4.8
Resident	996	83.6
Total	1191	

**Table 2:** FOCUS Quality Score Rubric

Collapsed Score	Raw Score	Raw Score Criteria	Collapsed Score
1	1	No Recognizable Structures, Insufficient for Diagnosis	Imaging Insufficient for Diagnosis
	2	Minimally Recognizable Structures, Insufficient for Diagnosis	

2	3	Structures Recognized with Some Technical Flaws, Minimal Criteria Met for Diagnosis	Minimum Imaging Criteria Met for Diagnosis

3	4	All Structures Imaged with Some Technical Flaws, Diagnosis Easily Supported	Imaging Easily Supports Diagnosis
	5	All Structures Imaged with Excellent Image Quality, Diagnosis Completely Supported	

*FOCUS*, focused cardiac ultrasounds.

**Table 3a:** Overall FOCUS Omission Rate by Patient Sex

FOCUS View	Female (n,%)	Male (n,%)	P value
PSL	75 (12.5%)	78 (13.0%)	0.80
PSS	122 (20.3%)	154 (25.7%)	0.03
A4C	215 (35.8%)	148 (24.7%)	<0.001
SX	252 (42.0%)	247 (41.2%)	0.77
IVC	552 (92.0%)	577 (96.2%)	0.002

*FOCUS*, focused cardiac ultrasounds; *PSL*, parasternal long axis; *PSS*, parasternal short axis; *A4C*, apical four-chamber; *SX*, subxiphoid; *IVC*, inferior vena cava.

**Table 3b:** Overall FOCUS Quality Score by Sex (total and by view)

	Female	Male	Total	p-value
	Mean (std)	Mean (std)	Mean (std)	
Total Score (0–20)	10.4 (3.5)	10.5 (3.5)	10.4 (3.5)	0.23
PSL (0–5)	2.9 (1.1)	2.9 (1.1)	2.9 (1.1)	0.58
PSS (0–5)	2.7 (1.2)	2.6 (1.3)	2.7 (1.2)	0.04
A4C (0–5)	2.3 (1.3)	2.6 (1.3)	2.5 (1.3)	<0.001
SX (0–5)	2.4 (1.5)	2.4 (1.5)	2.4 (1.5)	0.83

*FOCUS*, focused cardiac ultrasound; *PSL*, parasternal long axis; *PSS*, parasternal short axis; *A4C*, apical four-chamber; *SX*, subxiphoid; *IVC*, inferior vena cava.
