# Supplementary material for: Multicenter Study Evaluating Impact of Patient and Sonographer Demographics on Quality of Focused Cardiac Ultrasounds
Source: West J Emerg Med. 2025 Oct 3;26(5):1423–30. doi: 10.5811/westjem.38462 (PMC12591655; doi:10.5811/westjem.38462)
Supplement: Supplementary file 2 [file wjem-26-1423-s002.pdf]

Appendix 2

Linear Regression of Overall FOCUS Score & Patient Sex (unadjusted)		
**Variable**	**β-coefficient**	**P Value**
Patient Sex = Male	0.17	0.22
Reference: female		

Linear Regression of Overall FOCUS Score & Patient Sex (adjusted)		
**Variable**	**β-coefficient**	**P Value**
Patient Sex = Male	-0.01	0.92
Age 18-20	2.18	**0.01**
Age 21-44	1.05	**<0.01**
Age >65	-0.56	**<0.01**
BMI <18.5	-1.12	**0.01**
BMI 25-29.9	-0.30	0.13
BMI 30-34.9	-0.89	**<0.01**
BMI 35-39.9	-1.30	**<0.01**
BMI 40+	-2.17	**<0.01**
Operator Sex = Male	-0.60	**<0.01**
Operator Level = Att	-0.68	**0.01**
Operator Level = Fel	0.27	0.44
Reference Groups: age 45-65, operator sex female, operator level resident		
B-coefficients represent the expected change in overall FOCUS score on the 20 point scale between levels of each independent variable and the referent group. For example, in our unadjusted model (Table 4a), male patients had FOCUS scores 0.17 points higher than female patients		
