# Supplementary material for: Multicenter Study Evaluating Impact of Patient and Sonographer Demographics on Quality of Focused Cardiac Ultrasounds
Source: West J Emerg Med. 2025 Oct 3;26(5):1423–30. doi: 10.5811/westjem.38462 (PMC12591655; doi:10.5811/westjem.38462)
Supplement: Supplementary file 3 [file wjem-26-1423-s003.pdf]

Appendix 3

Logistic Regression A4C Collapsed Score (1-3) & Patient Sex (unadjusted)	
**A4C Score 2: OR (95% CI)**	**OR A4C Score 3: OR**
1.6 (1.3, 1.97)	1.68 (1.3, 2.16)

Reference Group: female

Logistic Regression A4C Score & Patient Sex (adjusted)		
**Variable**	**A4C Score 2: OR (95%**	**OR A4C Score 3: OR**
Patient Sex Male	1.69 (1.35, 2.12)	1.69 (1.28, 2.24)
BMI <18.5	1.18 (0.57, 2.44)	1.21 (0.54, 2.7)
BMI 25-29.9	1.12 (0.81, 1.54)	0.76 (0.53, 1.09)
BMI 30-34.9	0.68 (0.49, 0.93)	0.32 (0.21, 0.48)
BMI 35-39.9	0.52 (0.35, 0.78)	0.22 (0.13, 0.38)
BMI 40+	0.36 (0.23, 0.54)	0.2 (0.12, 0.35)
Age 18-20	0.76 (0.2, 2.96)	1.84 (0.49, 7.02)
Age 21-44	2.03 (1.5, 2.81)	2.14 (1.44, 3.17)
Age >65	1.24 (0.95, 1.61)	1.13 (0.81, 1.56)
Operator Sex Male	0.74 (0.58, 0.94)	0.68 (0.51, 0.91)
Operator Level Attending	0.66 (0.46, 0.96)	0.74 (0.46, 1.19)
Operator Level Fellow	1.38 (0.81, 2.34)	1.54 (0.8, 2.99)

Reference Group: A4C score 1, patient sex female, BMI 18.5-24.9, age 45-65, operator sex female, operator level resident
